# Supplementary material for: Mucosal tenofovir 1% gel stimulates cell proliferation and type I/III interferon pathways
Source: Microbiol Spectr. 2026 Mar 23;14(5):e01680-25. doi: 10.1128/spectrum.01680-25 (PMC13141841; doi:10.1128/spectrum.01680-25)
Supplement: Supplemental legends — Descriptive legends for Files S1 to S3. [file spectrum.01680-25-s0004.docx]

Legends for supplemental files

Supplemental file 1 – Gene fold changes comparing baseline to treatment within participant. Genes are identified by the “TargetId” and “EntrezId” columns. The “Significance” column identifies in which studies (if any) that gene had a significant change. The remaining columns are arranged in pairs, listing the log2 fold change and FDR-adjusted p-value for each study arm.

Supplemental file 2 – Results of gene set testing of the Hallmark gene sets. The “Gene_Set” column identifies the gene set. The remaining columns are arranged in pairs, listing the direction of change (where “Up” indicates higher during treatment compared to baseline) and FDR-adjusted p-value for each study arm.

Supplemental file 3 – Results of gene set testing of the Gene Ontology Biological Processes gene sets. The “Gene_Set” column identifies the gene set. The remaining columns are arranged in pairs, listing the direction of change (where “Up” indicates higher during treatment compared to baseline) and FDR-adjusted p-value for each study arm.
