# Supplementary material for: Mucosal tenofovir 1% gel stimulates cell proliferation and type I/III interferon pathways
Source: Microbiol Spectr. 2026 Mar 23;14(5):e01680-25. doi: 10.1128/spectrum.01680-25 (PMC13141841; doi:10.1128/spectrum.01680-25)
Supplement: Table S1 — ISG-24 gene set. [file spectrum.01680-25-s0005.docx]

| **Entrez ID** | **Gene Symbol** | **Gene name** |
| --- | --- | --- |
| 2537 | IFI6 | interferon alpha inducible protein 6 |
| 3429 | IFI27 | interferon alpha inducible protein 27 |
| 3434 | IFIT1 | interferon induced protein with tetratricopeptide repeats 1 |
| 3665 | IRF7 | interferon regulatory factor 7 |
| 4599 | MX1 | MX dynamin like GTPase 1 |
| 4600 | MX2 | MX dynamin like GTPase 2 |
| 4938 | OAS1 | 2'-5'-oligoadenylate synthetase 1 |
| 4939 | OAS2 | 2'-5'-oligoadenylate synthetase 2 |
| 4940 | OAS3 | 2'-5'-oligoadenylate synthetase 3 |
| 8519 | IFITM1 | interferon induced transmembrane protein 1 |
| 8638 | OASL | 2'-5'-oligoadenylate synthetase like |
| 9636 | ISG15 | ISG15 ubiquitin like modifier |
| 10379 | IRF9 | interferon regulatory factor 9 |
| 10410 | IFITM3 | interferon induced transmembrane protein 3 |
| 10561 | IFI44 | interferon induced protein 44 |
| 10964 | IFI44L | interferon induced protein 44 like |
| 11274 | USP18 | ubiquitin specific peptidase 18 |
| 51191 | HERC5 | HECT and RLD domain containing E3 ubiquitin protein ligase 5 |
| 54739 | XAF1 | XIAP associated factor 1 |
| 54809 | SAMD9 | sterile alpha motif domain containing 9 |
| 55008 | HERC6 | HECT and RLD domain containing E3 ubiquitin protein ligase family member 6 |
| 55601 | DDX60 | DExD/H-box helicase 60 |
| 91543 | RSAD2 | radical S-adenosyl methionine domain containing 2 |
| 122509 | IFI27L1 | interferon alpha inducible protein 27 like 1 |

**Supplemental Table 1: ISG-24 gene set.** A set of 24 interferon-stimulated genes affected by oral TDF/FTC.
